# Testosterone and Cortisol Jointly Predict the Ambiguity Premium in an Ellsberg-Urns Experiment

**DOI:** 10.3389/fnbeh.2017.00068

**Published:** 2017-04-21

**Authors:** Giuseppe Danese, Eugénia Fernandes, Neil V. Watson, Samuele Zilioli

**Affiliations:** ^1^Católica Porto Business School and CEGE, Universidade Católica PortuguesaPorto, Portugal; ^2^Neuropsychophysiology Lab, Centro de Investigação em Psicologia, Escola de Psicologia, Universidade do MinhoBraga, Portugal; ^3^Behavioral Endocrinology Laboratory, Department of Psychology, Simon Fraser UniversityBurnaby, BC, Canada; ^4^Department of Family Medicine and Public Health Sciences, Wayne State UniversityDetroit, MI, USA

**Keywords:** testosterone, cortisol, ambiguity, Ellsberg paradox, dual hormone hypothesis

## Abstract

Previous literature has tried to establish whether and how steroid hormones are related to economic risk-taking. In this study, we investigate the relationship between testosterone (T) and cortisol (C) on one side and attitudes toward risk and ambiguity on the other. We asked 78 male undergraduate students to complete several tasks and provide two saliva samples. In the task “Reveal the Bag,” participants expressed their beliefs on an ambiguous situation in an incentivized framework. In the task “Ellsberg Bags,” we elicited from the participants through an incentive-compatible mechanism the reservation prices for a risky bet and an ambiguous bet. We used the difference between the two prices to calculate each participant's ambiguity premium. We found that participants' salivary T and C levels jointly predicted the ambiguity premium. Participants featuring comparatively lower levels of T and C showed the highest levels of ambiguity aversion. The beliefs expressed by a subset of participants in the “Reveal the Bag” task rationalize (in a revealed preference sense) their choices in the “Ellsberg Bags” task.

## Introduction

Many papers study the relationship between hormones and economic risk-taking. Comparatively, fewer papers in behavioral endocrinology consider the fact that humans face different types of risk, and that these different types of risk might have different endocrine correlates. In economics and the management sciences, however, the distinction between risk proper and uncertainty (or ambiguity) has been customary ever since Knight (1921) first discussed the difference, followed decades later by Ellsberg (1961).

In one of Ellsberg's famous thought experiments, the decision maker can place bets on a black marble being drawn either from a bag with a known proportion of black and white marbles (the “risky” bag), or from a bag with unknown proportions (the “ambiguous” or “uncertain” bag). Once the participant chooses the bag, one marble is drawn. The color of the marble extracted determines whether the payoff is positive (if a black marble is drawn) or zero. Ellsberg speculated that decision makers would prefer to bet on the risky bag. He also speculated that this preference would likely hold regardless of the winning color (black or white), a finding confirmed in human and even in primate studies (cf. e.g., Hayden et al., 2010). These choices are inconsistent with the rational model of decision under uncertainty (Savage, 1972) and have given rise to many behavioral models that try to explain the preference for known-odds gambles (e.g., Gilboa and Schmeidler, 1989; Klibanoff et al., 2005; Seo, 2009).

The participants' ambiguity premium—the difference between the price the participants set to sell the bet on the risky bag minus the price they set for the bet on the ambiguous bag—provides a discrete measure of the strength of the participants' preference for known odds. A positive ambiguity premium is consistent with Ellsberg's insight that many participants might prefer known odds. A zero premium is consistent with a decision maker who does not differentiate between an equiprobable win or loss and complete lack of information about the chances of winning or losing. A negative premium implies a preference for ambiguous decisional situations. The most intuitive way to understand what kind of information the ambiguity premium conveys is the participants' willingness to pay to go from multiple possible scenarios about the content of the bag to one possible scenario only (equal probability of winning or obtaining zero).

Similarly to what has happened for other decisions that are inconsistent with economic theory, there has been an increasing effort to identify both the neural and hormonal correlates of anomalous behavior in risky and ambiguous situations. Functional magnetic resonance imaging studies have found that the representation of the subjective value of the risky and the ambiguous options seem to take place in the same area of the brain (the striatum and the medial prefrontal cortex; cf. Hsu et al., 2005; Levy et al., 2010). As for the role of hormones, the “dual-hormone hypothesis” (DHH) proposed that several types of human behaviors are explained by an interaction between Testosterone (T) and Cortisol (C). Mehta and Josephs (2010) suggest that status-seeking behaviors are to be expected among individuals with simultaneously high T and low C. The DHH seems to account for a growing number of results from studies on human aggressiveness, empathy, risk-seeking, status-seeking behavior, and overbidding in auctions (cf. Mehta and Prasad, 2015; Pfattheicher, 2017).

The theoretical forerunners of the DHH are the earlier studies that found that T correlated with aggressive behavior only in low-C offenders (Dabbs et al., 1991; Popma et al., 2007; Tackett et al., 2014). Many more papers studied the behavioral correlates of C only (levels or changes), of T only, or of both T and C, without controlling for the presence of interaction effects of C and T. Concerning C, low levels of this hormone were associated with fearlessness and reduced sensitivity to punishment and threats (Van Honk et al., 2003). On the other hand, high levels of C seemed to predict higher anxiety (Brown et al., 1996). T was found to be positively associated with dominance in social hierarchies, status-seeking behavior and success in competition both in animals and humans (the “challenge hypothesis,” cf. Mazur and Booth, 1998; Oliveira and Oliveira, 2014; Casto and Edwards, 2016; Wingfield, 2016).

The rationale for studying the endocrine correlates of *economic* risk is that risk-taking might have evolved as a way to increase status (Daly and Wilson, 1997; Ellis et al., 2012). Several studies have found a positive relation between risk-taking and T (cf., e.g., Apicella et al., 2008; Sapienza et al., 2009; Zilioli and Watson, 2012; but cf. the null results in Schipper, 2014; Cueva et al., 2015). Coates and Herbert (2008) found that traders in the City of London have significantly higher T levels on days when they made more than their 1-month daily average. The authors also found a strong positive correlation between the traders' daily C levels and the volatility of their net earnings on the day of the study. Van Honk et al. (2003) found that basal C negatively correlates with risky choices. Kandasamy et al. (2014) found that chronic (i.e., cumulative over several days) C exposure increased risk aversion. Mehta et al. (2015) found that basal testosterone is associated with higher financial risk-taking behaviors, but only for low C subjects, as predicted by the DHH.

Regarding the endocrine correlates of different types of risk, to the best of our knowledge T and C have not been addressed together in the same study. Stanton et al. (2011) found that neither the risk premium nor the ambiguity premium had a significant linear relationship with T (the predictor), and there were instead significant non-linearities in the relationship. Specifically, individuals that were risk and ambiguity averse were the ones who presented intermediate levels of T and individuals neutral to risk and ambiguity were at the two extremes of the distribution of T. Interestingly, their ambiguity task measured the participants' preferences between a situation of radical uncertainty vs. a situation of complete certainty. Their measure of the ambiguity premium is therefore not consistent with Ellsberg's thought experiment, i.e., a situation of known odds vs. unknown odds. Buckert et al. (2014) studied the relation between *C*, stress and decisions under risk and ambiguity. They found that after undergoing a stress induction protocol, the cortisol response did not affect the percentage of choices of the ambiguous option.

Any conclusion about the sign of the relationship between T and C and decisions under risk and ambiguity is complicated by the variety of tasks used in the literature (Schonberg et al., 2011); the differences between measuring circulating hormones from saliva, allocating participants to receive T (cf. e.g., Zethraeus et al., 2009, failing to find any effect of administering T on a variety of economic tasks) or proxying prenatal T by the 2D:4D finger ratio (Brañas-Garza and Rustichini, 2011); the sample used (the role of gender in particular, cf. e.g., Borghans et al., 2009). We re-examine the relation between T and C and ambiguity attitudes in Ellsberg's original framework. We use an incentive compatible elicitation mechanism to obtain a numeric measure of the participants' ambiguity premium. We design a novel task to elicit the beliefs of the players about an ambiguous situation, as these beliefs are not directly observable in Ellsberg-type experiments.

In line with previous behavioral economics research that links beliefs and choices (cf. e.g., Gilboa and Schmeidler, 1989), we expect to find a strong relationship between the beliefs of the players and their choices in the Ellsberg experiment. In addition, our design allows us to explore if the result in the literature concerning the positive association between T and risk-seeking behavior holds when different types of risks are involved. No previous study allows us to predict whether someone characterized by higher T would prefer knowing the odds and place a higher reservation price for the risky bet or prefer the ambiguous bet. It is also not clear *ex-ante* whether an ambiguity averse individual should exhibit endocrine correlates of higher stress, as we would expect based on some previous studies linking high C to pronounced risk aversion (Kandasamy et al., 2014; but cf. also Buckert et al., 2014). In our case, the outcome of both bets is unpredictable, and we lack the risk-free (degenerate) lottery that is often used to ascertain whether an individual is risk averse, risk seeking, or risk neutral. On the issue of the relationship between C and T, and their interaction, and the ambiguity premium, our analysis by necessity will be exploratory.

## Materials and methods

### Participants

Seventy-eight students participated in our experiments after they responded to a public announcement. The study was reviewed by the Office of Research Ethics of Simon Fraser University, and all participants provided written consent before the start of the experimental procedures. Exclusion criteria for the participation included (i) eating, drinking liquids other than water, smoking or brushing teeth in the hour prior to the session; (ii) consuming alcohol or drugs in the previous 12 h; (iii) intense physical activity on the day of the experiment; (iv) having a recent history of smoking more than 5 cigarettes a day, or of taking a medication that affects hormonal levels; (v) having bleeding gums and an oral infection. All participants were male undergraduate and graduate students of Simon Fraser University (mean age = 22.60, *SD* = 4.44, range = 18–42 years). The participants earned on average $19 Canadian during the experiment, the sum of their earnings in all the tasks they performed. Earnings were paid in cash at the end of the experiment. The entire experiment lasted on average 1 h 30 min.

### Procedure

The experiment took place in the afternoon (mean time: 2:30 PM, *SD* = 1 h 31 min). At the beginning of the session, participants completed a survey about their socio-demographic features as well as their recent health state. While completing the questionnaires, participants provided a salivary sample (see below). Afterward, they were tested in the three economic tasks explained in details in the next section (“Reveal the Bag” task or RB, “Ellsberg Bags” task or EB, “Monty Hall” task or MH). Tasks RB and EB were offered in random order, with 35% of the participants taking the RB task first. The MH task was always offered last. Instructions for all tasks are provided in the Supplementary Material (1) to the article available online. Tasks RB and EB were implemented without the help of computers, using bags filled with real marbles. The bags used were always randomly extracted from a shelf protected by a curtain visible to the participants, before they made their choices. This procedure was dictated by the desire to limit “malicious experimenter effects,” whereby the participant might believe that the experimenter (or the machine) filled the bags after having learned of the bets of the participants (cf. e.g., Kadane, 1992; Kühberger and Perner, 2003; Pulford, 2009). We provided the instructions of the tasks one at a time, and therefore subjects could not formulate at the beginning of the experimental session a strategy for each of the three tasks. To control for order effects of the tasks, we included a dummy for the order in which the RB task was offered as a robustness check (see below). Given the modest amounts of money at stake, we believe it is unlikely, but cannot ultimately exclude, that the amounts won in a previous task created an “endowment effect” (Kahneman et al., 1991) which affected the ensuing choices.

Interspersed with the three economic tasks, participants completed the BIS/BAS (Carver and White, 1994), Levenson's LSRP (Levenson et al., 1995) and Rotter's Internal-External Locus of Control Scale (Rotter, 1966) questionnaires. After all tasks and questionnaires were completed, participants provided a face picture and a scan of both their hands (not used for the analysis in this paper). Afterwards, a second salivary sample was collected. Subjects were then paid their earnings in each of the three tasks. The exchange rate was communicated in the instructions of each task (1 experimental point was always worth $0.20 Canadian). Subjects at this point left the experimental room.

### “Reveal the Bag” task (RB)

We devised this novel task to elicit the beliefs of the players about an uncertain situation. The experimenter presented to each participant a bag. Participants were informed that the bag contained 10 marbles, and each marble could be either white or black. Participants were asked to guess the bag's content. The experimenter randomly picked one bag from the shelf described above. Bags were replaced behind the curtain at the end of every participant's RB task. The extraction of the bag from behind the curtain was done in front of the participant.

Participants had 10 opportunities (or trials) to guess the composition of the bag and at the end of the 10th trial, they received a monetary reward according to the accuracy of their guesses. Each participant expressed his guesses on a white sheet that featured a printed grid of 11 columns and 10 rows (a sample sheet is reproduced in the SM). The rows represented the trials and the 11 columns represented all the possible scenarios for the composition of the bag, from all black to all white marbles. On each trial, the participant could bet on the bag composition by distributing 11 folder markers (small round stickers) along one row. When asked for the first time to guess the bag composition (first trial), the participant had no information regarding the black/white marbles ratio. After the participant had placed the 11 stickers in the first row, the experimenter started the second trial by extracting, without replacing, one marble from the bag. In the second row of the grid, the participant would write the color that had just been extracted and would express his new guess, by placing again 11 stickers on the sheet. On each trial, the experimenter revealed the color of one new marble. The task ended when the experimenter showed the color of the 10th marble, which completely revealed the composition of the bag. The monetary payoff was then calculated by summing the markers that the participant placed in the column that corresponded to the actual composition of the bag. In this way, we tried to ensure that the participants would use the available information and give some thought to the new information the experimenter provided about the content of the bag.

This task allows us to elicit, in an incentive-compatible framework, the beliefs of the subjects regarding an ambiguous situation that gradually becomes less ambiguous with the revelation of the color of the marbles. This task allows us to test whether the subjects' choices between the risky bag and the ambiguous bag in task EB, described next, are influenced by their beliefs about the contents of the ambiguous bag, as commonly assumed in behavioral models of ambiguity aversion (cf. e.g., Gilboa and Schmeidler, 1989; Klibanoff et al., 2005).

### “Ellsberg Bags” task (EB)

The participants were presented with two bags. The first bag contained 10 marbles, either white or black, in equal proportions. The content of this bag was shown to the participants. The second bag contained 10 marbles, either white or black, but in unknown proportions. The second bag was randomly chosen from the same shelf described in the RB task. The second bag was replaced on the shelf at the end of every participant's EB task.

Each participant was asked to choose a winning color for the two bags (white or black). The participant in this game had a right to extract a marble from each of the bags. If the marble extracted was of the same color that the participant has chosen, the participant won 15 experimental points. If the guess was incorrect, the participant won nothing. In an attempt to elicit the certainty equivalent for each lottery, the participants were asked to write two minimum selling prices for their two bets: *PA*, the price for the bet on the ambiguous bag, and *PR*, the price for the bet on the risky bag. The buyer of each bet was the experimenter, whose buying price for each bet was determined through a random physical mechanism (a number between 0 and 15 was drawn from yet another bag, with replacement). If the buying price for a bet was higher than, or equal to, the selling price stated by the participant, the participant pocketed the buying price, and no extraction took place from the corresponding bag. If the buying price was lower than the selling price the participant chose, the extraction of the marble took place. To ensure that the extraction of the random buying number for the first bet (the risky one) did not influence choices in the sale of the second bet, the experimenter's buying values were given only after the participant had stated both his prices. Instructions carefully explained that it was best for subjects to state the true value of the bets and that the price of the bets should reflect the desirability of the bets. If the subject thought the bet was very valuable, meaning that he thought the marble extracted would be very likely of the same color he chose, he should have stated a high selling price (close to 15). Conversely, a subject who believed that the bet was too close to call should have chosen a low price (close to zero), maximizing the chances that the bet would be bought by the experimenter at the random buying price. This elicitation procedure is known in experimental economics as the BDM method (Becker et al., 1964), and it was first adapted to the Ellsberg bags, to the best of our knowledge, by Halevy (2007). Further details on the pros and cons of the BDM method applied to lotteries can be found in Halevy's paper.

This task allows us not only to know if the participant is prey to the “Ellsberg paradox,” stating a higher price for the bet on the risky bag than for the bet on the ambiguous bag, but also to quantify each subject's aversion to ambiguity (or preference for ambiguity, if the price for the risky bag is lower than the price for the ambiguous bag).

### “Monty Hall” task (MH)

In the MH task participants were presented with three flipped cups and the experimenter stated that under one of the cups there was a black marble which could be exchanged for 15 experimental points. Participants were asked to indicate the cup they wanted to top flip. Next, the experimenter flipped one of the other cups, always one without any marble under it. The participants were then offered the possibility to stand by their initial choice of the cup to flip, or switch. This task is part of a project on the endocrine correlates of Bayesian updating, a topic we might study elsewhere, and with no hypothesized implication for the subjects' ambiguity attitudes studied here.

### Hormonal assays

Saliva samples were collected using Salimetrics Oral swabs (SOS; Salimetrics LLC, State College PA) placed under the tongue, according to vendor usage instructions for T determinations. According to the vendor, the SOS device consists of “an inert food-grade polymer” individually validated for use in specific assays that include salivary T and C determinations. Participants were instructed to place the oral swab beneath their tongue for at least 4 min. Samples were chilled immediately following collection, and then frozen within one h and held at −20°C until assay. Samples were assayed at the SFU Neuroendocrinology laboratory using competitive enzyme immunoassays for T and C (Salimetrics kits). For both steroids, the average intra-and inter-assay coefficients of variation were lower than 10%. The two samples provided by two participants were misplaced, and three participants were excluded due to them reporting in the demographics questionnaire that they were using medications (antibiotics hydrocortisone and medication for acne), leaving a final sample size of 73 participants. In all the statistical analyses used in this paper, we average the two measurements of T and C, to have a better proxy for the level of circulating hormones around the time of the experiment. Statistical tests presented in the SM show that differences in each participant's two measurements are not statistically significant.

## Results

### “Reveal the Bag (RB)” task

In this task, participants expressed their second-order beliefs regarding the contents of an ambiguous bag (10 marbles, either white or black). A second-order belief assigns a probability to a certain scenario for the ambiguous bag (e.g., six black marbles, four white marbles). We collected information about the second-order beliefs of the participants as we gradually revealed to them, marble after marble, the content of the bag.

**Figure 1:**
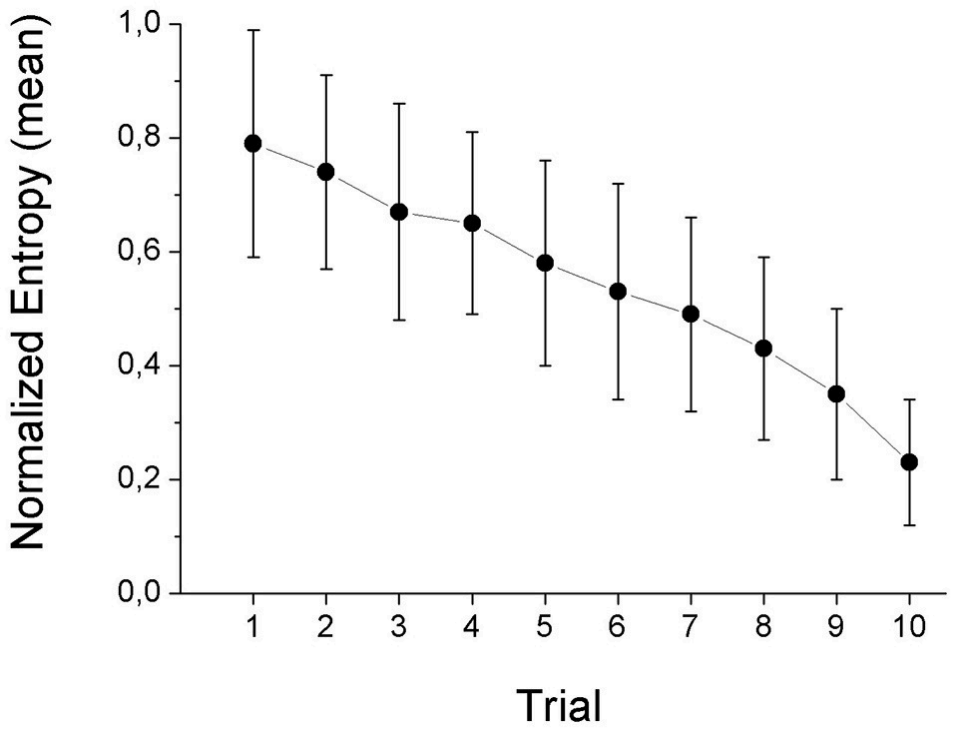
**Entropy of beliefs in the RB task**.

In an attempt to quantify the dispersion of beliefs about the content of the bag we computed an 11-bin histogram of all response possibilities. Afterward, we estimated the normalized entropy of the individual histograms, according to Shannon's formula (Shannon, 1948; Shannon and Weaver, 1949; cf. also Bennett et al., 2015):

$$\begin{array}{l} \text{H }=\;-\;\frac{\sum_{i=1}^{11}\left(p_{i}\;\times \;\log_{2}p_{i}\right)}{\log_{2}11\;} \end{array}$$

where *p*_*i*_ is the relative frequency at bin *i*. The normalized entropy reflects the degree of belief uncertainty regarding the bag composition. The H scores range from 0—when the second-order probability mass lies all on one scenario (e.g., six white and four black), to 1—when all scenarios (from all-black to all-white) are believed to be equally likely. The mean entropy (over participants) from trial 1 (the color of zero marbles has been revealed, complete ambiguity) to trial 10 (only the color of one marble remains to be revealed) is shown in Figure 1. The downward trend in the dispersion of beliefs was clear. Entropy followed a constant rate of decay. To confirm this finding, we regressed average entropy in each trial on the trial number, finding a significant negative relation (*p* < 0.001). About one-quarter of the participants in the first trial had an entropy of 1, i.e., they thought all the scenarios were equally likely (the so-called Laplace Principle of Insufficient Reason, cf. Gilboa, 2009, p. 14). Only 3% of the participants thought there was only one possible scenario for the bag (entropy of zero). The remaining cases fell in between (cf. Figure 1 and the histograms in the SM). In about 8% of the total number of cases the participants committed a mistake, by placing positive probability mass on scenarios that were ruled out by the available information (e.g., attributing positive probability to the “10 black, 0 white marbles” after one white marble had already been revealed to them). This provides evidence that subjects understood the task and considered the information that was presented to them in order to make their choices.

### “Ellsberg Bags (EB)” task

Table 1 shows the descriptive statistics for PR, PA, and the ambiguity premium (PR-PA).

**Table 1:** **Descriptive statistics of the EB task**.

**Variable**	**Obs**	**Mean**	**Std. Dev**.	**Min**.	**Max**.
PR	73	7.87	2.87	0	15
PA	73	7.27	2.81	0	13
Premium (PR-PA)	73	0.59	2.52	−6.5	8

The reservation prices of the two lotteries were close, which resulted in an ambiguity premium of small positive magnitude, consistent with a modest degree of ambiguity aversion in the sample. The modal choice of premium is zero (31.5% of the subjects). As in Stahl (2014), we find that ambiguity preferences are heterogeneous and that a high degree of aversion to ambiguity might not be the most common finding, as instead the earlier literature supposed (cf. e.g., Halevy, 2007). Both a paired *t*-test and a non-parametric Wilcoxon signed-rank test rejected the null that the average of *PR* is equal to the average of *PA* (*p*-value is in both cases <0.05). The two prices were very close to the expected value of the bet on the risky bag, i.e., 7.5 points (regardless of the color chosen). The fact that PR was on average above the expected value of the lottery implies that participants were on average modestly risk-loving, as in Halevy (2007). When we calculated the average premium as a percentage $\frac{\left(\bar{PR}-\bar{PA}\right)}{\bar{PA}}\times 100$, the result, 7.5%, was well-below the figure reported in Halevy (2007), i.e., 20%. Borghans et al. (2009), who also used the BDM design, reported a percentage figure of around 15%. The proportion of participants who were ambiguity neutral is comparable to Halevy's finding (current study: 30%; Halevy: 22%). PA and PR were positively correlated (*r* = 0.61): this implies that typically participants displayed either a general distaste for seeing the realization of their random bets (when they chose low prices for the two lotteries) or a general taste for seeing the realization of their bets.

### Regression and interaction analysis

We used regression analysis to determine whether centered average T (${\overset{-}{t}}_{i}-\overset{-}{t}$), centered average C (${\overset{-}{c}}_{i}-\overset{-}{c}$) and the interaction term between the two average centered hormonal measurements predicted our dependent variable *y* (the ambiguity premium). Each ${\overset{-}{t}}_{i}$ (${\overset{-}{c}}_{i}$) is the average of the participant's two T (C) measurements (cf. also the SM for robustness checks using only the first measurement). The regression model is shown in Equation 1 (*i* is the identifier of the participant).

(1) $$\begin{array}{r} y_{i}=\alpha +\gamma \left(\left[{\overset{-}{c}}_{i}-\overset{-}{c}\right]\right)+\delta \left(\left[{\overset{-}{t}}_{i}-\overset{-}{t}\right]\right)+\theta \left(\left[{\overset{-}{c}}_{i}-\overset{-}{c}\right]\right)*\left(\left[{\overset{-}{t}}_{i}-\overset{-}{t}\right]\right)+\varepsilon_{i} \end{array}$$

The reason for subtracting the mean across participants of the average hormonal measurements ($\overset{-}{t}$ and $\overset{-}{c}$) from each individual's average measurement, a procedure known as “centering,” was that, when using uncentered variables, average C and T were highly correlated with the T^*^C interaction term, creating a multicollinearity problem. Aiken and West (1991) suggested centering as a solution to this issue, and the variance inflation factor for the interaction term went from 48 in the uncentered model to 1 in the centered model. Table 2 shows the regression output of regression model (1), estimated through Ordinary Least Squares, with robust standard errors (R-squared = 0.063, model is significant at 5%).

**Table 2:** **Linear regression predicting the ambiguity premium based on centered hormones**.

**Dependent variable: premium**	**Coef**.	**Robust Std. Err**.
AvgC_centered	−1.939	2.972
AvgT_centered	−0.009	0.009
CrossCT_centered	0.179^***^	0.067
Constant	0.413	0.309

*** *p ≤ 0.01*.

The interaction term between T and C had a significant, positive relation with the ambiguity premium. Several robustness checks presented in the SM confirm this finding. We also show in the SM that the significance of the interaction term in Table 2 can be probably attributed to the strong relation between the two hormones and PA.

**Figure 2:**
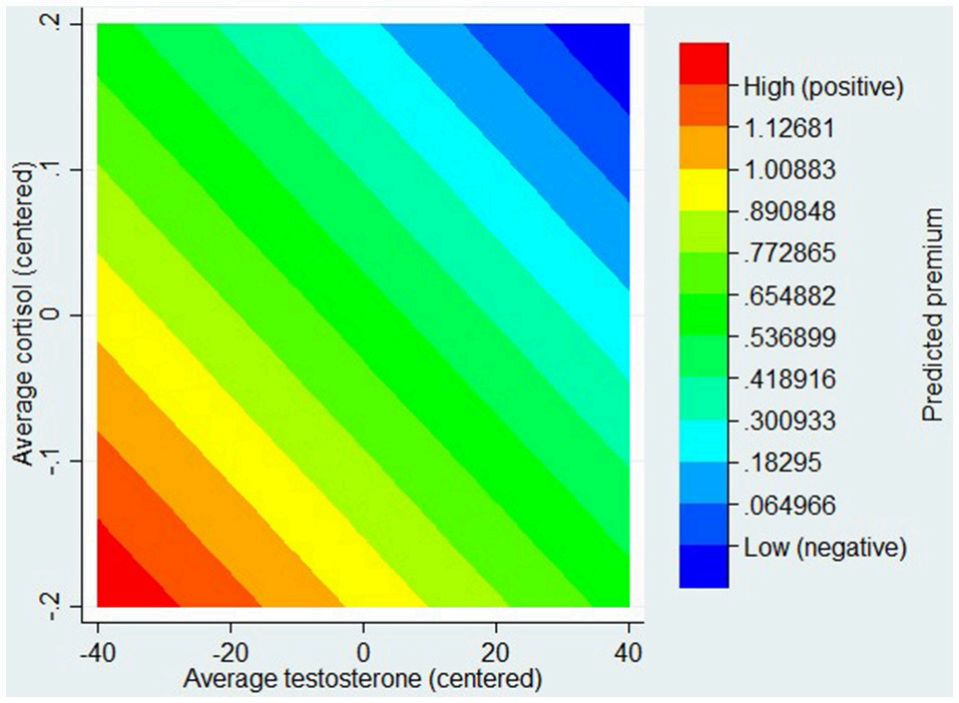
**Contour plot of the predictive margins of regression model (1)**.

The positive sign of the interaction term, together with the negative signs of T and C, implied an overall negative relationship between the two hormone levels and the ambiguity premium. In Figure 2, we show a contour plot of the predictive margins of the regression model (1). On the axes, we plot T and C about two standard deviations below and above the (zero) mean. The color bands show different levels of (predicted) premium. The Figure shows that a group of participants, specifically those with comparatively lower levels of T and C, exhibited comparatively higher aversion to ambiguity. The aversion to ambiguity declined as T increased, both for the low C and the high C group. The significance of the interaction term in Table 2 ensures that this pattern is statistically significant.

### Beliefs and ambiguity attitudes

We used the responses of the participants in the first trial of the RB task to analyze the choice of reservation prices for the lotteries in the EB task. The participants were not given any signal that the ambiguous bag used in the EB task was the same as the ambiguous bag in the RB task. Given that, however, we were in the realm of complete uncertainty, it seems likely that the participants might have held the same beliefs regarding the ambiguous bag they faced in the first trial of the RB task and the ambiguous bag in the EB task. Using a revealed preference approach, *PR* would be greater than *PA* if the Expected Utility of the lottery defined over the risky bag (*EU*(*L*_*R*_)) was greater than the (Subjective) Expected Utility of the lottery defined over the ambiguous bag (*SEU*(*L*_*A*_)). Details of the expected utility calculations for the two lotteries are given in the SM.

The average across participants of the difference between the expected utilities of the two lotteries, which we call π (π = [*EU*(*L*_*R*_)−*SEU*(*L*_*A*_)]) is positive (the estimate is $\tilde{\pi }=\;0.094$). Together with the finding that the ambiguity premium in the EB task is on average positive, this finding shows that participants found on average bets on a risky bag more attractive than bets on an ambiguous bag. For the participants with entropy equal to 1 in the first trial of the RB task the expected utility of the two EB lotteries was the same, and $\tilde{\pi }=0$ was the modal estimate. Together with the finding that the modal value for premium is zero, these two results show that neutrality was the most common attitude to ambiguity in our experiment. We then carried a direct comparison between each participant's π_*i*_ and his ambiguity premium. These are two different ways to express the desirability of the bet on the risky bag vs. the bet on the ambiguous bag. Thirty-two percent of the participants passed this test of coherency, i.e., the sign of the variable premium is the same as the sign of π, or they are both zero. Of particular interest is a group of participants, 16% of the total, who featured a $\tilde{\pi }=0$ and also a premium equal to zero. These participants expressed their neutrality to ambiguity in a remarkably consistent way. We found no evidence that hormones played a role in determining the responsiveness of the choices of the risky lottery to π in a softmax model like the one used by Frydman et al. (2011). Finally, we do not find any role of T and C in explaining the degree of entropy of the participants' choices in trial 1 of the RB task.

## Discussion

We established that there were instances in which the beliefs of the players translated into choices of one bet vs. the other. Moreover, we found cases in which subjects had an ambiguity premium equal to zero *and* derived, in our armchair calculations that involved some parameter and functional form choices, the same expected utility from the two bets (risky and ambiguous). This congruency is to be expected if the beliefs of the players about the bags are related to their ambiguity attitudes, as we hypothesized. Yet this congruency is not assured for most participants, contrary to our expectations. Possible reasons are that we used responses from two different tasks in our expected utility computations, assuming that the beliefs in round 1 of the RB task were the same as the beliefs about the ambiguous bag in the EB task, an assumption that might not be valid for all participants. Another possible explanation is that some parameter and functional form choices had to be made *ex-ante* and we did not build around the expected utility estimates an interval that allows for perceptual mistakes about the lotteries and the bags.

We found a significant interaction effect between T and C and the ambiguity premium in an Ellsberg experiment. The participants displaying the highest premium were those with lower C and lower T. These participants showed a preference for known odds of winning compared to ignorance about the odds. This preference attenuated as cortisol and testosterone jointly increased. This finding supports *some* aspects of the DHH. This hypothesis has two parts: one is methodological, in the sense that it recommends that regression models using C and T should also control for the interaction effects of the two hormones, a suggestion we use and which yields some insights into the endocrine correlates of risk and ambiguity. The second part of the DHH is substantive, and it posits that T should positively correlate with status-seeking behavior only in low C individuals. No consensus exists on the substantive claim (cf. e.g., Welker et al., 2014, finding that testosterone is positively related to aggression only for high C individuals), and we do not find evidence in its favor. The substantive claim of Mehta and Josephs (2010) is, however, not easily applicable to our design, given the presence in our study of both risk *and* ambiguity. The part of behavioral endocrinology concerned with economic risk-taking is most likely not impermeable to “garden of forking paths” issues (Gelman and Loken, 2014), a problem that might be due to the low number of observations in some studies, affecting the power of the statistical testing procedures. We have tried to ease this problem writing pre-analysis plans (for the beliefs part of the analysis) while acknowledging where our analysis becomes exploratory due to the novelty of the study.

Unlike in Stanton et al. (2011), we did not find any evidence of a non-linear relation between T and the ambiguity premium (cf. also the robustness checks in the SM and earlier work by Schipper, 2014). Comparisons between the results of Stanton et al. (2011) and ours are, however, complicated by the differences in the design.

T has been associated to outperforming in competitions and to status-seeking behavior (Zilioli and Watson, 2014). It could play a role in ambiguous decisions involving monetary gains because most competition situations are ambiguous, in the sense that beliefs about the skills and threat posed by the opponent might be difficult to formulate (cf. Oliveira and Oliveira, 2014, on cognitive appraisal of competitive situations). We would expect therefore participants with higher levels of T to prefer situations that are more ambiguous and potentially more rewarding, displaying a lower premium, as shown in our study. C is the end product of the hypothalamic–pituitary–adrenal stress axis (Dedovic et al., 2009). Higher C might be related to higher sensitivity to stressors in the decision context, and therefore it seems sensible that individuals with high trait T (who preferred ambiguous situations) might also be characterized by higher levels of C. In the current study ambiguity only surrounded the probability of winning (rather than e.g., also the probability of losing, or smaller vs. greater gains), and a high T individual might have preferred the ambiguous bag out of confidence that the ambiguous bag offered higher-probability gains than the risky one. It is left for future research to establish if T and C are positively correlated with a preference for ambiguity when ambiguity entails potentially bigger gains compared to the risky situation. A question we have not addressed is gender-effects in ambiguity and risk attitudes (cf. e.g., Borghans et al., 2009; Lighthall et al., 2009; Boksem et al., 2013; Kandasamy et al., 2014; Schipper, 2014). Future studies might ask whether our results from a male population extend also to females.

We contribute new evidence to the behavioral endocrinology literature, in particular the branch that focuses on choices over lotteries and their link to T and C. This field has to this date not converged to a consensus about the significance and direction of either T or C, or both, for risk-taking behavior, a situation that invites new studies and replication of existing ones. We hope future research will also bear in mind Ellsberg (1961)'s remark that “not all risks are the same” when discussing risk-taking and its hormonal correlates.

## Ethics statement

This study was carried out in accordance with the recommendations of the Ethical Conduct for Research Involving Humans guidelines (TCPS-2), Office of Research Ethics of Simon Fraser University, with written informed consent from all subjects. All subjects gave written informed consent in accordance with the Declaration of Helsinki. The protocol was approved by the Office of Research Ethics of Simon Fraser University.

## Author contributions

GD conceived of the study, drafted the manuscript, and carried out the statistical analyses. EF helped draft the manuscript, participated in the statistical analysis. SZ participated in the design of the study, coordinated the study, and supervised the hormonal analyses. NW provided advice and facilities for the hormonal analyses. All authors gave final approval for publication.

## Funding

We wish to acknowledge a Teaching and Learning Development Grant from Simon Fraser University (number L-G0029) to GD and a Discovery Grant 0194522 from the Natural Sciences and Engineering Research Council of Canada (NSERC) to NW.

### Conflict of interest statement

The authors declare that the research was conducted in the absence of any commercial or financial relationships that could be construed as a potential conflict of interest. The reviewer PR and handling Editor declared their shared affiliation, and the handling Editor states that the process nevertheless met the standards of a fair and objective review.
