# Supplementary material for: Testosterone and Cortisol Jointly Predict the Ambiguity Premium in an Ellsberg-Urns Experiment
Source: Front Behav Neurosci. 2017 Apr 21;11:68. doi: 10.3389/fnbeh.2017.00068 (PMC5399068; doi:10.3389/fnbeh.2017.00068)
Supplement: Supplementary file 1 [file DataSheet1.docx]

Supplementary Material

Testosterone and Cortisol Jointly Predict the Ambiguity Premium in an Ellsberg-urns Experiment

Giuseppe Danese*, Eugénia Fernandes, Neil V. Watson, Samuele Zilioli

*** Correspondence:** Giuseppe Danese: gdanese@porto.ucp.pt

**Descriptive statistics**

Table 1-SM shows the descriptive statistics that are not shown in the main article, for the behavioral tasks, questionnaire scores, and the two hormonal measurements.

**Table 1-SM.**

Descriptive statistics

|  |  |  |  |  |  |  |  |
| --- | --- | --- | --- | --- | --- | --- | --- |
|  | **Variable** |  | **Obs** | **Mean** | **Std. Dev.** | **Min** | **Max** |
|  |  |  |  |  |  |  |  |
| **Tasks** |  |  |  |  |  |  |  |
|  |  |  |  |  |  |  |  |
| EB |  |  |  |  |  |  |  |
|  | BlackR |  | 78 | 0.45 | 0.50 | 0 | 1 |
|  | BlackA |  | 78 | 0.40 | 0.49 | 0 | 1 |
|  |  |  |  |  |  |  |  |
| RB |  |  |  |  |  |  |  |
|  | H_1_^st^ |  | 76 | 0.79 | 0.20 | 0 | 1 |
|  | Prop.Errors |  | 701 | 0.08 | 0.32 | 0 | 3 |
|  |  |  |  |  |  |  |  |
| MH | Bayes |  | 77 | 0.57 | 0.50 | 0 | 1 |
|  |  |  |  |  |  |  |  |
|  |  |  |  |  |  |  |  |
| **Questionnaires** |  |  |  |  |  |  |  |
|  |  |  |  |  |  |  |  |
| BIS/BAS |  |  |  |  |  |  |  |
|  | BIS_tot_ |  | 77 | 20.36 | 3.54 | 12 | 28 |
|  | BAS_tot_ |  | 77 | 39.08 | 4.28 | 30 | 51 |
|  | BAS_drive_ |  | 77 | 10.78 | 2.08 | 6 | 16 |
|  | BAS_fun_ |  | 77 | 11.31 | 2.01 | 6 | 16 |
|  | BAS_reward_ |  | 77 | 16.99 | 2.25 | 11 | 20 |
|  |  |  |  |  |  |  |  |
| LSRP |  |  |  |  |  |  |  |
|  | LSRP_tot_ |  | 77 | 65.62 | 5.17 | 55 | 77 |
|  | LSRP-I |  | 77 | 40.21 | 3.56 | 34 | 50 |
|  | LSRP-II |  | 77 | 25.42 | 3.12 | 20 | 32 |
|  |  |  |  |  |  |  |  |
| Rotter |  |  |  |  |  |  |  |
|  | Rotter_tot_ |  | 76 | 11.43 | 2.41 | 6 | 19 |
|  |  |  |  |  |  |  |  |
| **Hormones** |  |  |  |  |  |  |  |
|  |  |  |  |  |  |  |  |
| Cortisol | C_t0_ |  | 73 | 0.17 | 0.11 | 0.02 | 0.73 |
|  | C_t1_ |  | 73 | 0.17 | 0.16 | 0.03 | 0.96 |
|  |  |  |  |  |  |  |  |
| Testosterone | T_t0_ |  | 73 | 154.64 | 31.19 | 89.96 | 237.63 |
|  | T_t1_ |  | 73 | 157.61 | 30.54 | 97.44 | 244.20 |
|  |  |  |  |  |  |  |  |

BlackR and BlackA are the percentage of participants who decided to bet on black on the risky and ambiguous bags, respectively. There is no clear sign of a “color” preference for betting on black versus white, with perhaps a slight bias towards betting on white.

H_1_^st^ is the mean normalized entropy in the first trial of the RB task (Figure 1-SM). We also report the proportion of mistakes committed in the course of the RB task, when subjects placed stickers on scenarios that were clearly impossible given the available information. About 60% of the subjects are able to use Bayes rule in the MH task, again a signs that reassures about our participants’ ability to work with probabilities.

| **A.** Examples of distributions of responses with their respective H scores | |
| --- | --- |
| H = 0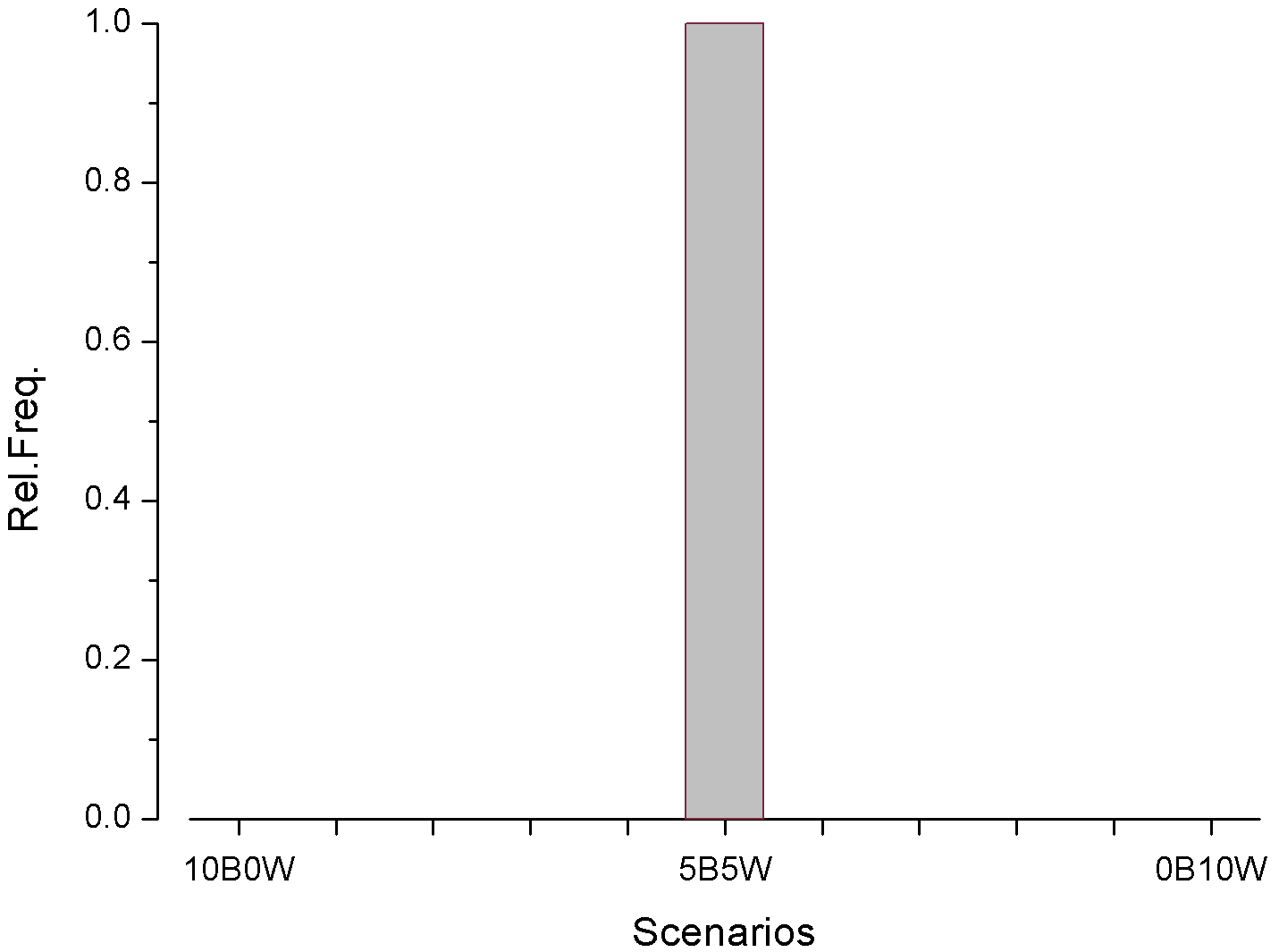 | H = 0.287  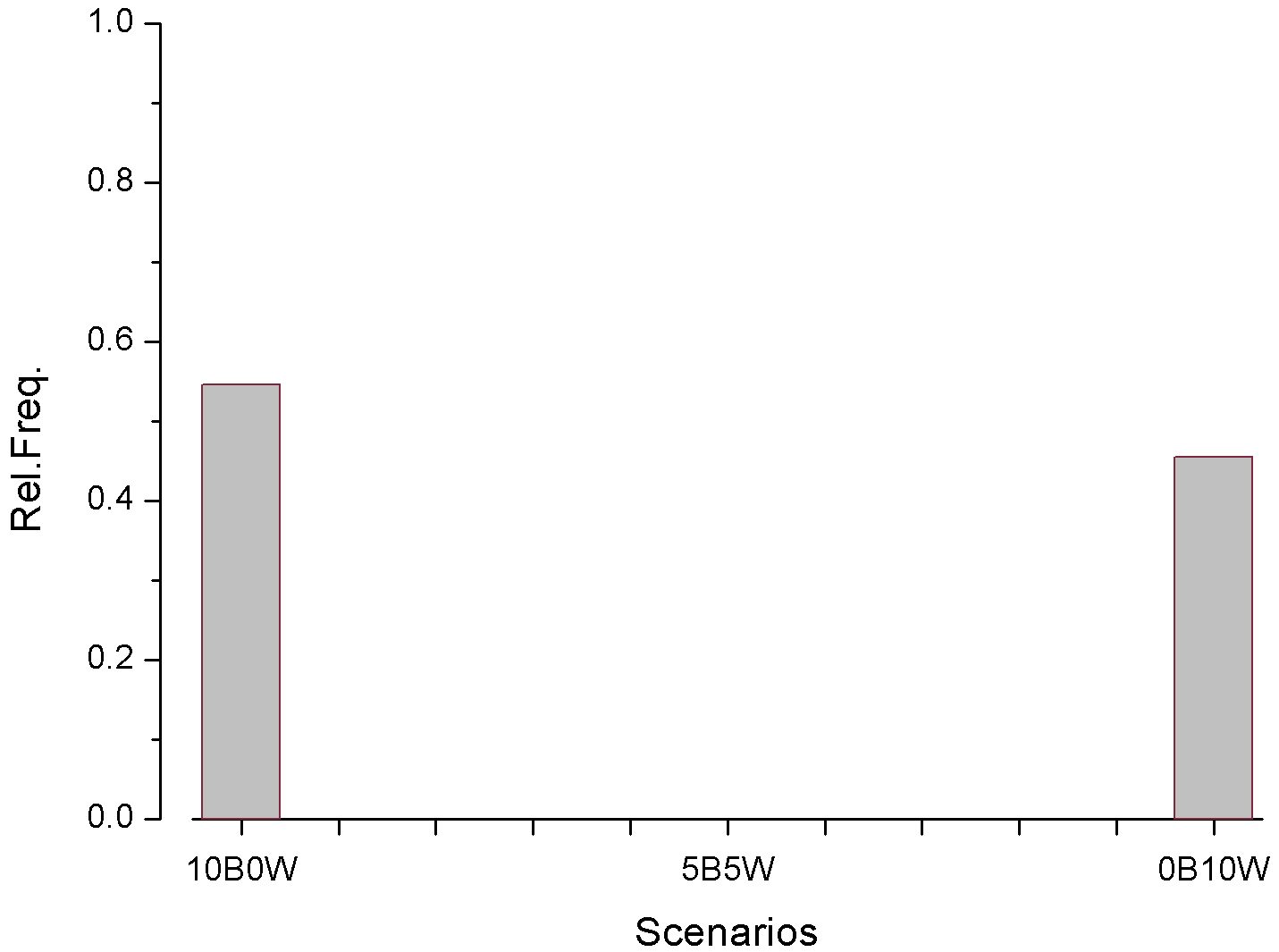 |
| H = 0.612  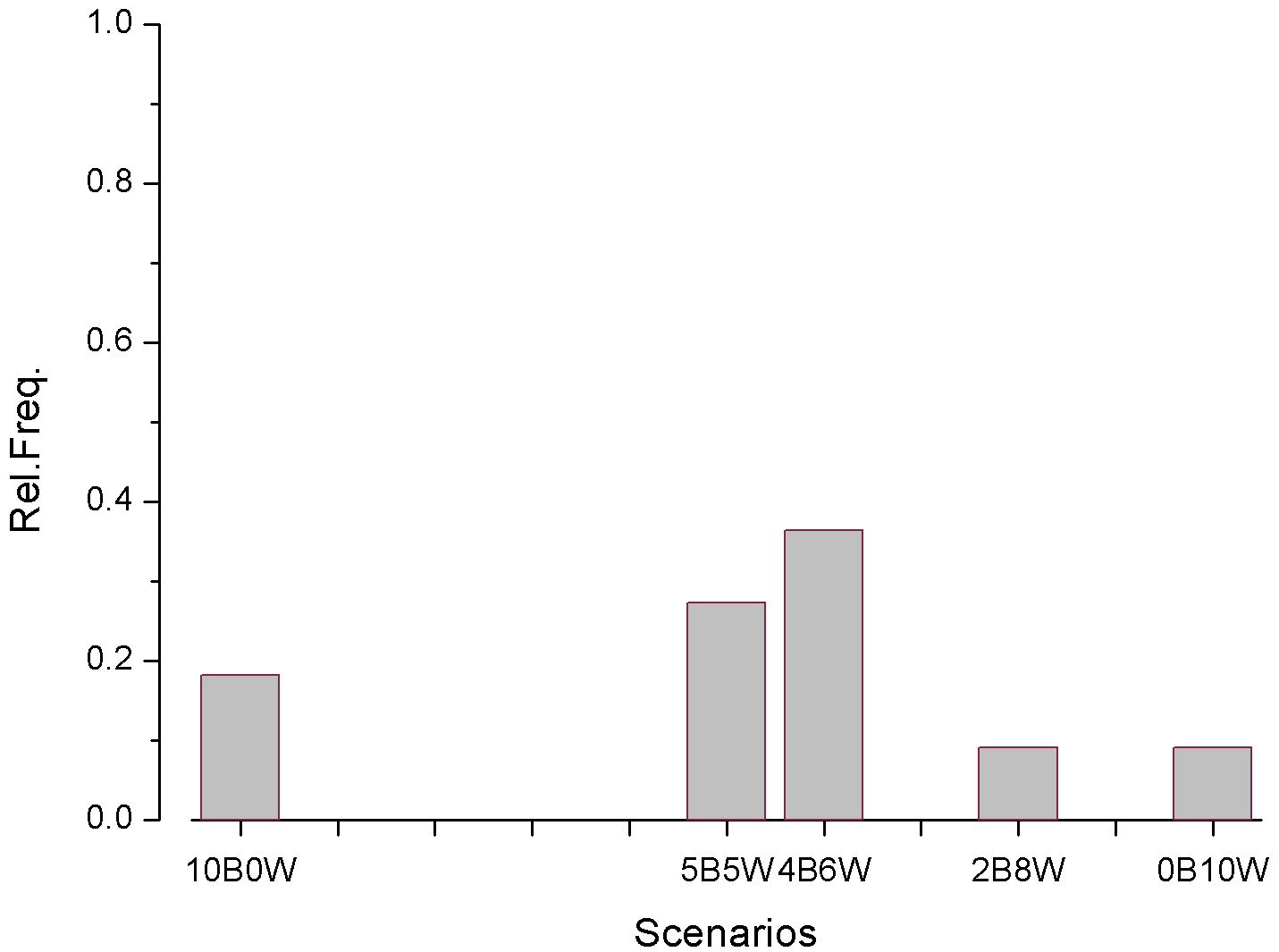 | H = 1  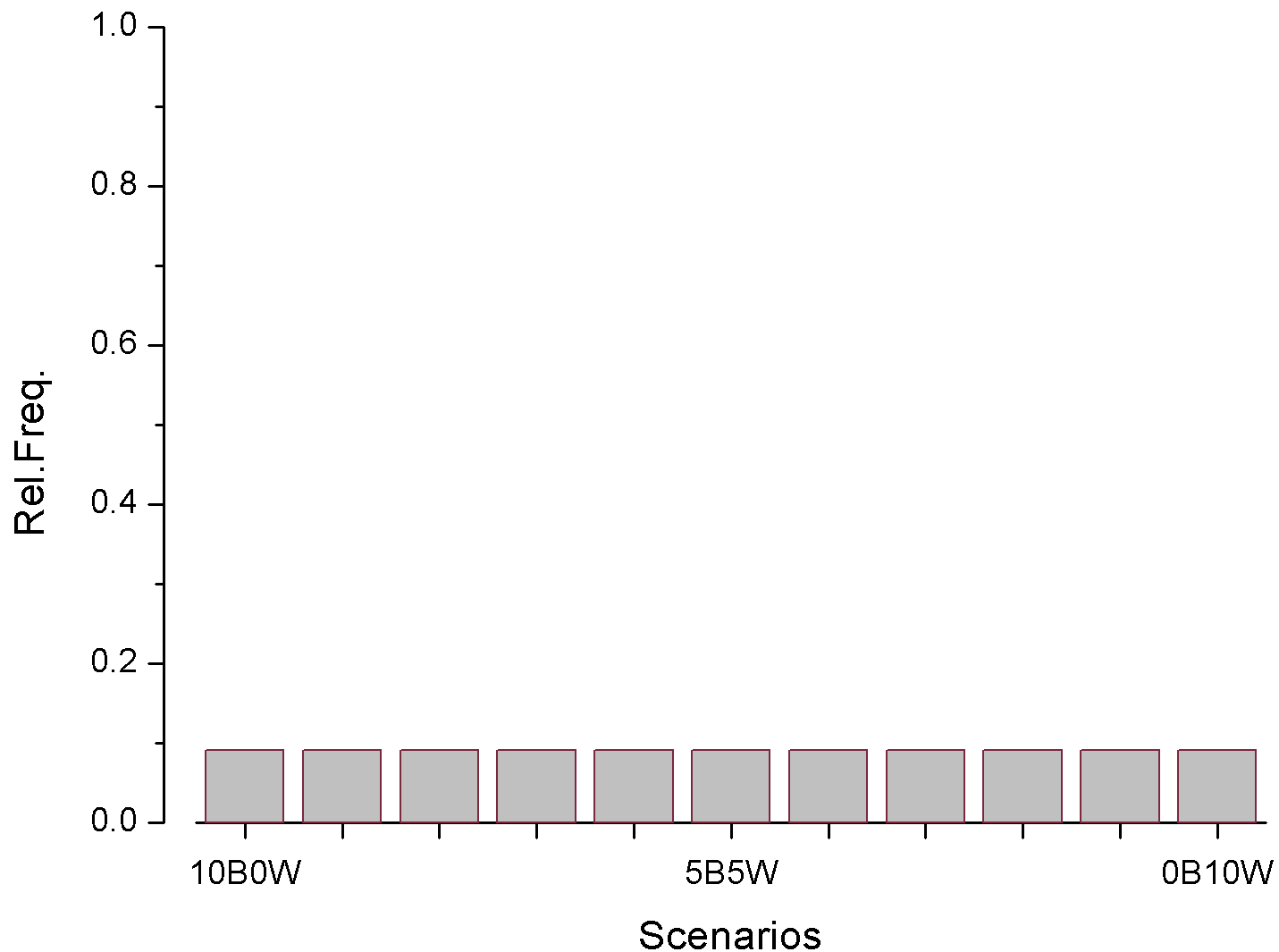 |
|  |  |
| **B.** Histogram of Entropy scores during first trial (variable H_1_^st^) | |
| 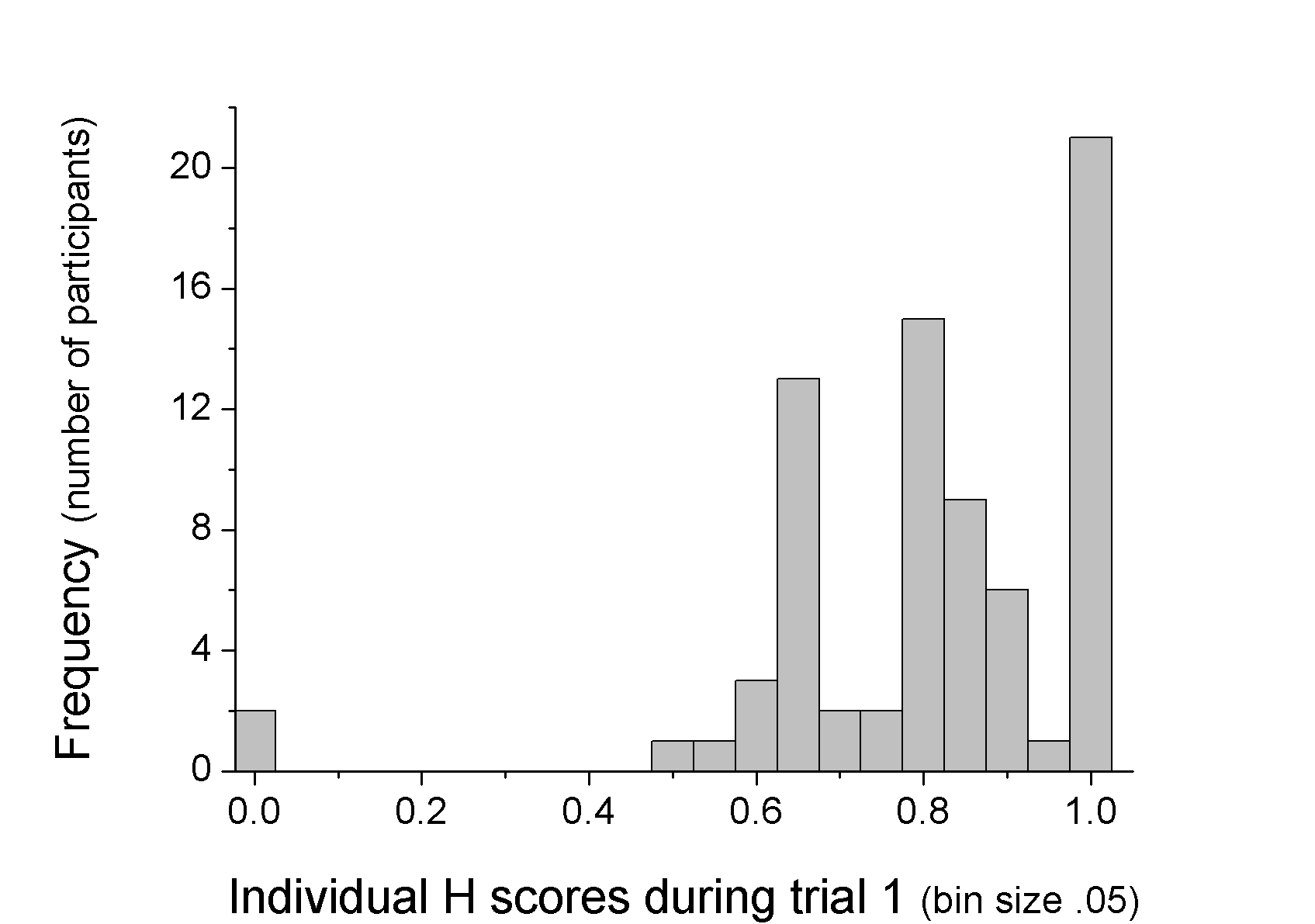 | |

**Figure 1-SM.** **(A.)** Four examples of distributions of responses and their respective H scores. Each graph represents a hypothetical first trial of the RB task, where subjects placed 11 stickers along the eleven possibilities of the bag composition (from 10 blacks and 0 whites to 0 blacks and 10 whites). **(B.)** Histogram of all subject’s entropy scores during the first trial of the RB task.

The BIS/BAS questionnaire is composed of one behavioral inhibition scale (BIS) and three behavioral activation (BAS) subscales: drive, fun seeking and reward responsiveness. In the original study by Carver & White (1994), the sub-sample of 358 male college students presented mean scores close to those we found in our study. Respectively, males in Carver & White’s study presented BIS scores with M = 18.84, BAS_reward_ with M = 17.27, BAS_drive_ with M = 12.05, and BAS_fun_ with M = 12.43.

The LSRP-I and LSRP-II subscales evaluate primary (callous, uncaring, selfish and manipulative attitudes) and secondary (impulsivity and emotional instability) psychopathy traits. The maximum possible raw scores are of 64 and 40 for the LSRP-I and LSRP-II, respectively. In the original study by Levenson *et al.* (1995), the authors report separate mean scores for men and women (about 133 men), but no measures of dispersion. Our sample’s primary and secondary psychopathy mean scores are comparatively higher than those originally reported by Levenson *et al*. (LSRP-I mean = 32.96, LSRP-II mean = 20.04). In comparison with other studies of male non-offenders (i.e., noninstitutionalized populations), the psychopathy trait scores in the current study were also found to be higher (Ferrigan *et al.*, 2000; Ross *et al.*, 2004; Elwood *et al.*, 2004; Walters *et al.*, 2009; Marion & Sellbom, 2011; Gummelt *et al.*, 2012). We suggest that further studies should inspect the impact of variables such as cultural background, age and academic course (e.g., most noninstitutionalized samples are composed of undergraduate psychology students) in LSPR scores.

The Rotter questionnaire may yield a raw score ranging from extreme internal (0) to extreme external (23) locus of control. In the original study of Rotter (1966), mean scores from sub-samples of males from different populations (e.g., universities) ranged from M = 6.06 with SD = 3.51 (N =122) to M = 10.00 with SD = 4.20 (N = 32). Several studies have since focused on cultural differences. For instance, Smith *et al.* (1995) compared mean scores of 6332 males from 43 countries. Mean externality scores from males across the 43 countries ranged from M=6.35 (72 Pakistani), to M=12.69 (134 formerly eastern Germans). Although on the upper side, the average scores in our study fall within the values found in this cross-cultural study. Comparison with our data is hindered, though, because there was no subsample of Canadian males. Moreover, large variability seems to occur within the same culture. For instance, a curious finding was that while they presented very low scores in the 1995 study, a more recent study by Zaidi & Mohsin (2013) found that Pakistani graduation students (N=100, aged 18-25 years) presented a mean externality score of 11.32 (SD=2.29). Of notice, such values are close to those found in our study.

Hormones were measured at the beginning (t_0_) and the end (t_1_) of each session. A paired samples 2-tailed t-test revealed no significant difference between the two measurements of cortisol (t(72) = -.244, p = .808) and of testosterone (t(72)=-1.379, p = .172). This justifies the choice in the experiment to average the two measurements.

To inspect the relationship between personality measurements and C and T levels, we conducted both Pearson (Table 2-SM) and Spearman (omitted) correlation analyses. We found no significant relation between personality measurements (i.e., self-report questionnaire scores) and hormonal levels. There was only a significant, negative correlation between age and testosterone (as in Harman et al., 2001, and Ellison et al., 2011). It is possible that our self-report instruments did not successfully probe for those personality traits thought to be involved in the relationship between hormones and externalizing problems (e.g., Tackett *et al*., 2014; Yildirim & Derksen, 2015; Loomans *et al*., 2015; Pfattheicher, 2016).

**Table 2-SM.**

Pearson correlations based on the untransformed (average) values of T and C

|  |  |  |  |  |  |
| --- | --- | --- | --- | --- | --- |
|  |  |  | **Pearson correlation coefficient** | | |
| **Variable** |  |  | **Testosterone** |  | **Cortisol** |
|  |  |  |  |  |  |
| **Age** |  |  | -.28* |  | .02 |
|  |  |  |  |  |  |
| **Tasks** |  |  |  |  |  |
| RB | H_1_^st^ |  | .14 |  | -.02 |
| **Questionnaires** |  |  |  |  |  |
| BIS/BAS |  |  |  |  |  |
|  | BIS_tot_ |  | .01 |  | .04 |
|  | BAS_tot_ |  | -.07 |  | -.10 |
|  | BAS_drive_ |  | -.15 |  | -.04 |
|  | BAS_fun_ |  | .04 |  | -.08 |
|  | BAS_reward_ |  | -.03 |  | -.08 |
|  |  |  |  |  |  |
| LSRP |  |  |  |  |  |
|  | LSRP_tot_ |  | -.02 |  | -.16 |
|  | LSRP-I |  | -.05 |  | -.12 |
|  | LSRP-II |  | .02 |  | -.13 |
|  |  |  |  |  |  |
| Rotter | Rotter_tot_ |  | -.03 |  | .01 |
|  |  |  |  |  |  |

* p < .05

**Robustness checks**

**The ambiguity premium as the dependent variable**

We first present some robustness checks for the centered model we use in the paper, and then we move to the uncentered model. In the paper (Table 2) we show a significant interaction term in predicting the premium. Figure 2-SM illustrates the interaction between T and C in predicting premium as in Aiken & West (1991). This figure, as the contour plot shown in the paper, shows that the ambiguity premium decreases as C increases, both for the low T and high T individuals.

**Figure 2-SM.**

Simple slope analysis as in Aiken & West (1991)

The literature often transforms cortisol into its logarithm to correct for skewness in the hormonal data. To check whether a transformation of the hormonal variables is needed, we use the method first suggested by Box & Tidwell (1962). The procedure yields a p-value for each regressor, where the null hypothesis is that the regressor should *not* be transformed nonlinearly. We fail to reject the null for the two steroids, even when we include in the test the interaction terms and controls. Introducing the square of testosterone in the analysis, for comparison with Stanton *et al*. (2011), leaves the signs, magnitudes of the coefficients and p-values reported in the paper unaffected. As expected from the results of the Box & Tidwell (1962) procedure, the square of testosterone is insignificant, again a confirmation that there is no need to transform T nonlinearly in our model.

A concern in the choice of estimation method is that the dependent variable (the ambiguity premium) features a large number of zeros (about 31.5% of the sample, cf. Figure 3-SM).

**Figure 3-SM.**

Histogram of the ambiguity premium.


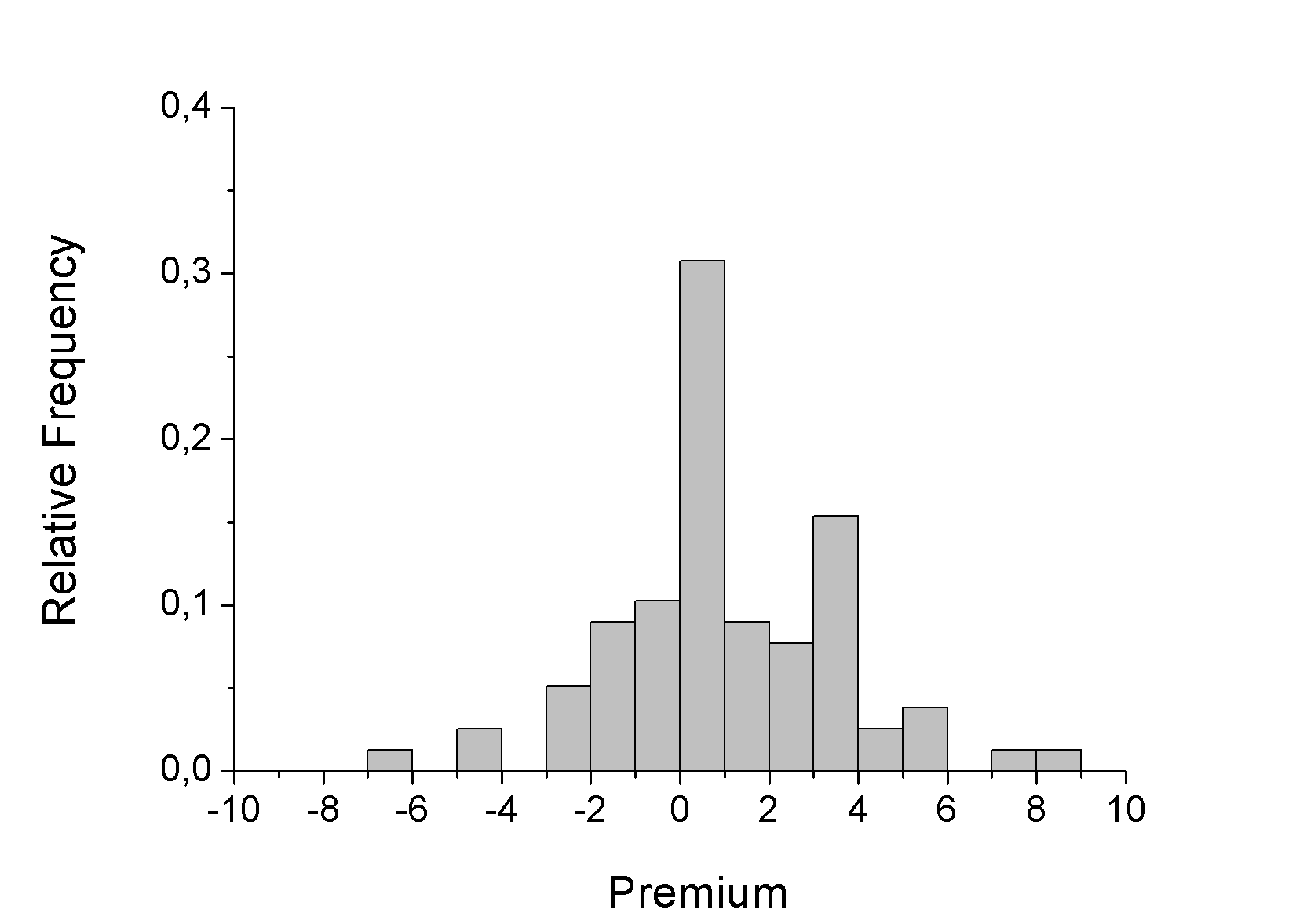


We use a nonlinear ordered multinomial logit analysis to check the robustness of the effect of the interaction term. The model is “ordered” because a natural grouping method arises. All subjects with a negative premium are group 1, all subjects with a zero premium are group 2, and all the subjects with a positive premium are group 3. The model does not include an intercept, as the latent dependent variable *y** crosses two thresholds, which can be estimated (cut1 and cut2 in Table 4-SM, cf. Cameron & Trivedi, 2005, p. 519 and ff.). In the ordered logit model, the error term is assumed to be logistic distributed. The sign of the parameters of interest for testosterone and cortisol tells us whether the latent dependent variable *y** increases or decreases with cortisol and testosterone. The interpretation of the coefficients is complicated by the nonlinear estimation method. The regression output with robust standard errors and centered variables are shown in Table 4-SM.

**Table 4-SM.**

Logit regression predicting the ambiguity premium based on centered hormones

|  |  |  |  |
| --- | --- | --- | --- |
| Regressors |  | Coef. | Robust Std. Err. |
|  |  |  |  |
| AvgC_centered |  | -2.02 | 2.82 |
| AvgT_centered |  | -0.009 | 0.006 |
| CrossCT_centered |  | 0.17** | 0.08 |
| cut1 |  | -0.95 | 0.28 |
| cut2 |  | -0.48 | 0.27 |
|  |  |  |  |

Note: ** p < .05

Again our analysis finds a significant positive relationship between the interaction term of testosterone and cortisol and the ambiguity premium. Using only the first measurement (t_0_) of testosterone and cortisol in a linear model (with centered hormones) again yields a significant positive interaction term (Table 5-SM).

**Table 5-SM.**

Linear regression predicting the ambiguity premium based on centered hormones (first measurement only)

|  |  |  |  |
| --- | --- | --- | --- |
| Regressors |  | Coef. | Robust Std. Err. |
|  |  |  |  |
| C_t0__centered |  | -0.2 | 2.53 |
| T_t0__centered |  | -0.01 | 0.01 |
| CrossC_t0_T_t0__centered |  | 0.09*** | 0.03 |
| Constant |  | 0.5 | 0.3 |

Note: *** p < .01

Augmenting the centered model with: order of tasks, entropy in the first trial of the RB task, age in years, a dummy for Asian ethnicity, time of collection of the first saliva sample, scores from BIS, BAS, LSRP, and Rotter questionnaires, ability to perform Bayesian updating (from the MH task) leads to coefficients of the same sign and comparable magnitudes to the estimates presented in the paper (Table 6-SM).

**Table 6-SM.**

Linear regression predicting the ambiguity premium based on centered hormones and controls

|  |  |  |  |
| --- | --- | --- | --- |
| Regressors |  | Coef. | Robust Std. Err. |
|  |  |  |  |
| AvgC_centered |  | -2.849 | 3.115 |
| AvgT_centered |  | -0.013 | 0.010 |
| CrossCT_centered |  | 0.194*** | 0.063 |
| Reveal_first |  | 0.503 | 0.775 |
| RB_ H_1_^st^ |  | 0.001 | 0.002 |
| MH_Bayes |  | -0.511 | 0.627 |
| Age |  | -0.044 | 0.087 |
| Asian |  | -0.304 | 0.628 |
| BAS_tot_ |  | -0.105 | 0.090 |
| BIS_tot_ |  | -0.135 | 0.081 |
| LSRP_tot_ |  | -0.044 | 0.074 |
| Rotter_tot_ |  | -0.113 | 0.182 |
| Time |  | 0.000 | 0.000 |
| Constant |  | 12.012 | 5.963 |
|  |  |  |  |

Note: *** p < .001

We now present the results of the regression analysis using the *uncentered* testosterone and cortisol values. We first estimate the model through OLS, with robust standard errors (Table 7-SM). The Box-Tidwell procedure tells us again that there is no need to transform nonlinearly cortisol or testosterone.

**Table 7-SM.**

Linear regression predicting the ambiguity premium based on uncentered hormones

|  |  |  |  |
| --- | --- | --- | --- |
| Regressors |  | Coef. | Robust Std. Err. |
|  |  |  |  |
| AvgC |  | -29.86** | 12.70 |
| AvgT |  | -0.04*** | 0.01 |
| CrossCT |  | 0.18** | 0.07 |
| Constant |  | 6.97 | 2.47 |

Note: *** p < .01, ** p < .05.

The signs of the coefficients of the regressors of interest (testosterone, cortisol, and the cross-term) are the same as in the centered model. In the uncentered model testosterone and cortisol are also significant, as well as the cross-term, whose p-value is unaffected by centering. As we mention in the paper, the model suffers from multicollinearity issues.

When we estimate the uncentered model through logit (Table 8-SM), we obtain again the same signs as in the model that uses OLS.

**Table 8-SM.**

Logit regression predicting **Premium** based on uncentered hormones

|  |  |  |  |
| --- | --- | --- | --- |
| Regressors |  | Coef. | Robust Std. Err. |
|  |  |  |  |
| AvgCort |  | -27.98** | 13.23 |
| AvgTest |  | -0.04*** | 0.01 |
| CrossTerm |  | 0.16** | 0.07 |
| /cut1 |  | -7.16 | 2.16 |
| /cut2 |  | -5.73 | 2.11 |
|  |  |  |  |

Note: *** p < .01, ** p < .05.

The model using only the first sample of testosterone and cortisol once again produces the same signs and similar p-values for the coefficients of interest. The interaction analysis in the uncentered model using OLS shows that the group of participants who is most averse to ambiguity is the group with low testosterone and low cortisol. This is the same result discussed in the paper for the centered model. The premium declines as testosterone increases, as in the analysis presented in the paper. The relation between premium and cortisol is instead more nuanced in the uncentered model: the predicted premium decreases as cortisol increases, as in the analysis presented in the paper, but the relation breaks for values of the dependent variable close to zero or negative (Figure 4-SM).

**Figure 4-SM.**

Contour plot of predictive margins with the ambiguity premium as the dependent variable and uncentered homones.


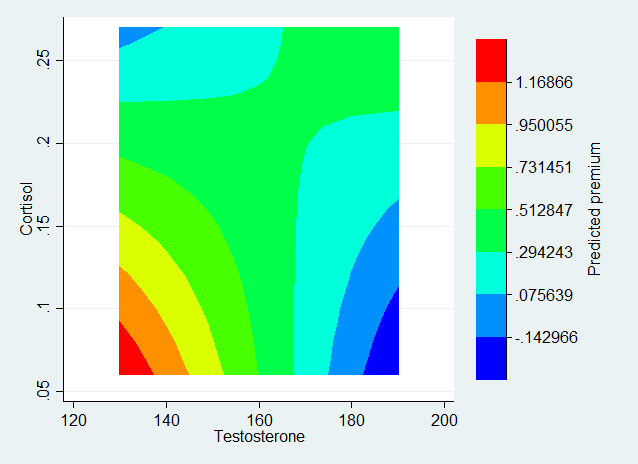


**PR, PA and risk premium as the dependent variable**

Borghans *et al.* (2009) run the regressions separately for PR and PA, suggesting that two prices might have different correlates. PR and PA in themselves are not useful, however, to infer the level of ambiguity or risk aversion of the participants. For completeness, we report the regression output of model (1) in the paper with PR (Table 9-SM) and PA (Table 10-SM) as the dependent variables, respectively.

**Table 9-SM.**

Linear regression predicting PR (reservation price for the risky bet) based on centered hormones

|  |  |  |  |
| --- | --- | --- | --- |
| Regressors |  | Coef. | Robust Std. Err. |
|  |  |  |  |
| AvgC_centered |  | 0.68 | 3.30 |
| AvgT_centered |  | 0.00 | 0.01 |
| CrossCT_centered |  | -0.08 | 0.09 |
| Constant |  | 7.95 | 0.34 |

**Table 10-SM.**

Linear regression predicting PA (reservation price for the ambiguous bet) based on centered hormones

|  |  |  |  |
| --- | --- | --- | --- |
| Regressors |  | Coef. | Robust Std. Err. |
|  |  |  |  |
| AvgC_centered |  | 2.61 | 2.46 |
| AvgT_centered |  | 0.01 | 0.00 |
| CrossCT_centered |  | -0.26*** | 0.06 |
| Constant |  | 7.53 | 0.33 |

Note: *** p < .01.

The analysis finds a negative relationship between PA and the interaction term. For most cortisol values, higher testosterone is associated with a higher price for the ambiguous urn. This finding, as already remarked, does not yield any information about the hormonal correlates of the ambiguity attitude of the participants, which can only be measured when PA is subtracted from PR. The results of the analyses on PR and PA suggests that our result regarding the relation between the ambiguity premium and the interaction term of T and C is likely driven by the price the subjects set for the ambiguous urn. Although PR, on its own, is not a dependent variable of interest to this study, the lack of correlation with the interaction term is puzzling. Reasons might include the particular task that we used, sampling, or some more fundamental flaws in the DHH that might lead to a “garden of forking paths” problem (Gelman & Loken, 2014), discussed in the paper.

In a previous study of risk and ambiguity attitudes under induced stress Buckert *et al*. (2014) found no effect of cortisol response on the percentage of choice of the ambiguous option. The findings in this study that C and T have an effect on the premium and on the price of the ambiguous lottery will hopefully motivate a re-examination of the link between ambiguity, stress, and cortisol.

A regression of the *risk* premium, calculated as the difference between the expected value of the risky lottery (7.5 points) and the certainty equivalent (PR), on centered hormones and controls yields no significant correlations.

**Details of expected utility calculations**

Using a revealed preference approach, $PR>PA$ if $EU(L_{R})>SEU(L_{A})$, where PR and PA are, as usual, the certainty equivalent of the risky lottery ($L_{R})$ and of the ambiguous lottery ${(L}_{A}$). Given that the lottery defined over the risky bag $L_{R}$ gives 15 and 0 with equal probability, $EU(L_{R})$=$0.5*u\left( g \right)+0.5*u\left( 0 \right)$, where g is the gain (15 points). We use here the utility function of prospect theory for gains (Tversky & Kahneman, 1991), namely $u\left( x \right)=g^{0.88}$. This utility function is concave, taking a reference point of zero. We do not distort the probabilities, as studies have found little distortion around the 0.5 probability. For our particular risky lottery, therefore, there are no differences between a traditional von Neumann-Morgenstern expected utility calculation and a prospect theory calculation.

It is typically impossible to obtain $SEU(L_{A})$ because the subjects’ beliefs about the ambiguous bag cannot be inferred from prices in Ellsberg-type experiments. The first trial of the RB task gives us the second order-probabilities (of all the possible scenarios, from all black to all white) that make the calculation possible. Let us take for example a subject who has placed a bet on a black marble being extracted from the ambiguous bag in the EB task. For the sake of illustration, let us suppose that the subject exhibits maximum entropy in his/her beliefs at trial 1 of the RB task, i.e. he thinks that all the possible scenarios for the composition of the bag in the RB task are equally likely. As we report in the paper, this type of belief about the scenarios is the modal belief in our experiments. Crucially, let us assume that the participant has the *same* beliefs about the contents of the ambiguous bag in the RB task (trial 1) and in the EB task. In this case:

$SEU\left( L_{A} \right)=p_{10b}\left[ (1*u\left( g \right)+0*u(0) \right]+p_{9b}\left[ (\frac{9}{10}u\left( g \right)+\frac{1}{10}*u(0)) \right]+p_{8b}\left[ (\frac{8}{10}u\left( g \right)+\frac{2}{10}*u(0)) \right]+p_{7b}\left[ (\frac{7}{10}u\left( g \right)+\frac{3}{10}*u(0)) \right]+p_{6b}\left[ (\frac{6}{10}u\left( g \right)+\frac{4}{10}*u(0)) \right]+p_{5b}\left[ (\frac{5}{10}u\left( g \right)+\frac{5}{10}*u(0)) \right]+p_{4b}\left[ (\frac{4}{10}u\left( g \right)+\frac{6}{10}*u(0)) \right]+p_{3b}\left[ (\frac{3}{10}u\left( g \right)+\frac{7}{10}*u(0)) \right]+p_{2b}\left[ (\frac{2}{10}u\left( g \right)+\frac{8}{10}*u(0)) \right]+p_{1b}\left[ (\frac{1}{10}u\left( g \right)+\frac{9}{10}*u(0)) \right]+p_{0b}\left[ (0*u\left( g \right)+1*u(0)) \right]$ (1SM)

Outside the parentheses are the second-order probabilities the subjects express in the RB task (trial 1). These second-order probabilities are “objective”, i.e. we do not distort them to account for behavioral regularities such as the overweighting of low-probability scenarios. Inside the parentheses are the objective probabilities of winning, given the (undistorted) second order probability. We assume the decision maker reduces the first-stage of the lottery (where the composition of the bag is determined) and the second-stage (where a marble is randomly extracted) to one lottery through multiplication (cf. Halevy, 2007, on the reduction of compound lotteries axiom of expected utility and its implications for ambiguity attitudes). The SEU calculation presented here, therefore, does not take into account any anomaly in the reduction of compound lotteries or non-linear weighting of probabilities.

If we specify, as in the case of the risky lottery, a parametric form for the utility function ($u\left( x \right)=g^{0.88}$), expression (1SM) yields a real number, that is expressed in the same unit as $EU\left( L_{R} \right),$“utils”. We compute $SEU\left( L_{A} \right)$ for each subject, given the second-order probabilities expressed in the RB task, and objective probabilities of winning and losing given by the color the subject has chosen for his bet on the ambiguous bag.

**APPENDIX 1**

**Copy of an RB sheet**


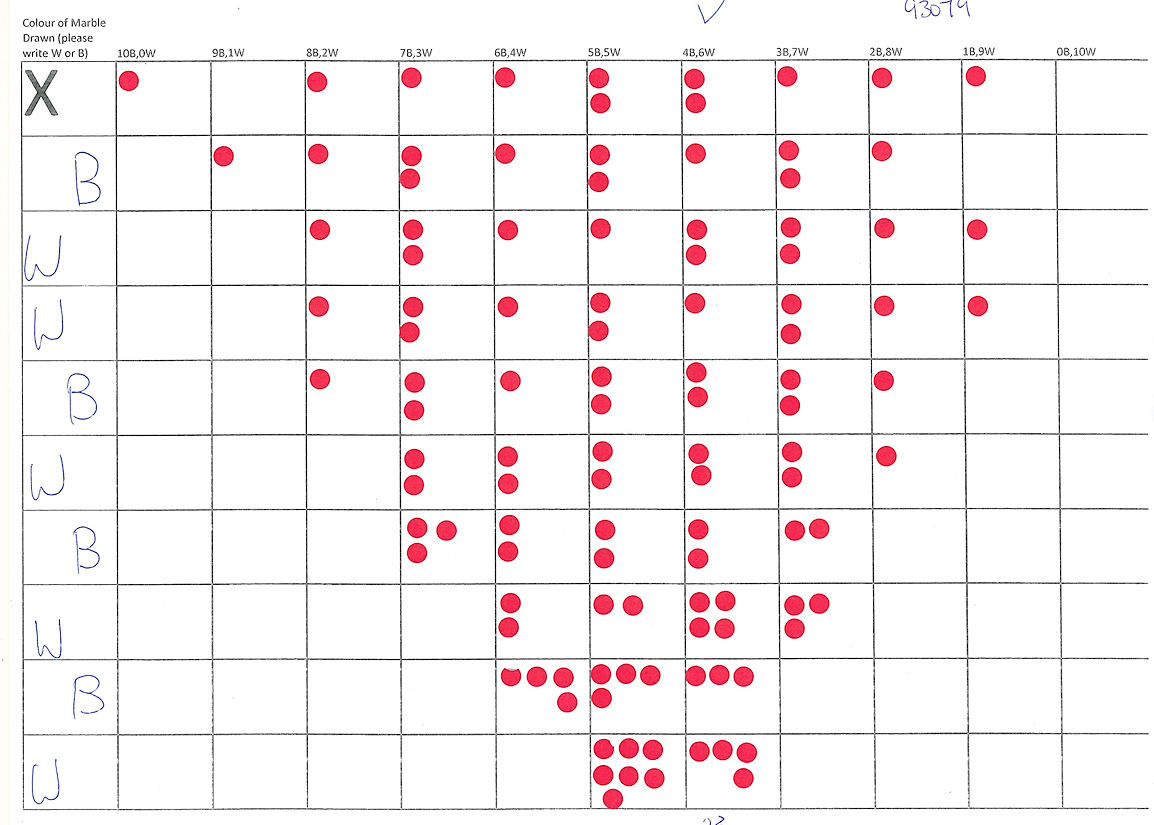


**APPENDIX 2**

**Copy of an EB sheet**


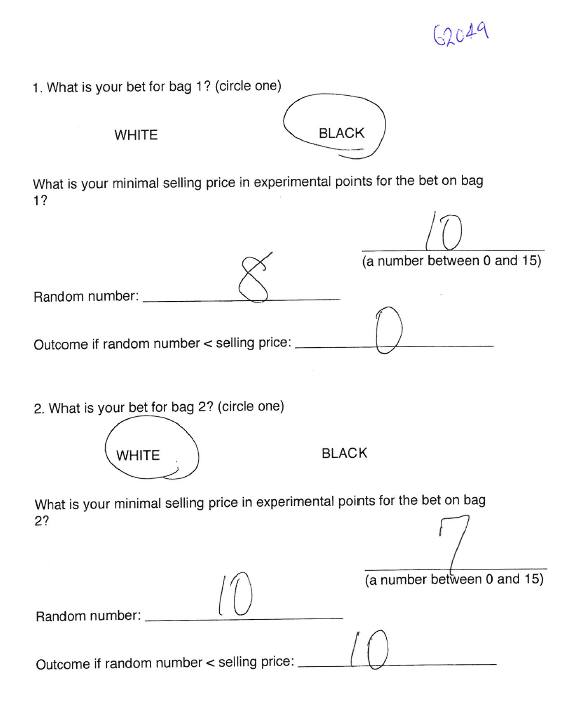


**APPENDIX 3**

**Instructions for task RB**

In front of you there is a bag containing ten marbles (small balls). The marbles are colored, either white or black. The experimenter will reveal to you, one at a time, the color of all the marbles in the bag. Before revealing each marble, your task is to place 11 stickers in a row corresponding to the number of marbles revealed up to that point on the sheet in front of you. These stickers represent your guesses about the number of white and black marbles in the urn. You can place in every cell of a given row any number of stickers between 0 and 11. Exactly 11 stickers need to be placed in each row.

As soon as the color of the last marble is revealed, we will sum the stickers you placed in the column corresponding to the true composition of the urn. That number will be your payoff in experimental points. Every experimental point is worth $0.20.

Any question?

**APPENDIX 4**

**Instructions for task EB**

*NB: The instructions used in this task replicate closely those used in Halevy’s paper, provided by the author in an on-line appendix. This ensures maximum comparability of the results.*

In front of you there are two bags, each containing 10 marbles, which can be either white or black. The composition of the two bags is as follows:

Bag 1: Contains 5 white marbles and 5 black marbles.

Bag 2: 10 marbles, either white or black

You are asked to place two bets on the color of the marble drawn from each bag. You can bet on different colors for the two bags.

Before marbles are drawn from each bag, you may sell each one of your bets. You are asked to state two minimal prices at which you are willing to sell each of the two bets. The two minimal prices have to be between 0 and 15 experimental points. For each bag, a random number between 0 and 15 will be generated. The two random numbers will be the buying prices in experimental points for each one of the bets. If the buying price for a bag is higher than the minimal selling price you stated for that bag, you will be paid the buying price (and will not have to wait for the outcome of your bet). However, if the buying price for the bag is lower than the minimal selling price you stated for that bag, your payment will depend on the outcome of your bet. If your guess is correct, i.e. the marble drawn from each bag is of the same color you have placed your bet on, you win 15 experimental points. If your guess is incorrect, you win nothing.

Note that it is in your best interest not to overstate your selling price for the two bets because this lowers the chances you will be able to sell your bet and does not increase the buying price. Likewise, it is in your best interest not to understate your selling price for the two bets, because this may force you to sell a bet at a price that is lower than your valuation of the bet.

For example, suppose you want to sell a $1 coin you have. Clearly, its value is exactly $1. If you state a selling price higher than $1 (say $1.50), you might not be able to sell it even if the buying price is as high as $1.49—a profitable transaction. Likewise, if you state a selling price lower than $1 (say $0.75), you might be forced to sell your coin at a loss (if the buying price is between $0.75 and $0.99). The only way you are sure not to lose is if you state a selling price of exactly your valuation ($1 in this case). It is important that your selling prices reflect how attractive each bet is: the more attractive it is for you to participate in a bet, the higher the selling price you should state.

Every experimental point is worth $0.20.

**APPENDIX 5**

**Instructions for task MH**

In front of you there are three cups. Hidden under one of these cups there is a black marble. If you find the black marble, you will be paid a prize of 15 experimental points. Every experimental point is worth $0.20.

Which cup would you like to flip?

‘’’

Which cup would you like to flip?

**References – SM**

Borghans, L., Golsteyn, B.H.H., Heckman, J.J., & Meijers, H. (2009). Gender differences in risk aversion and ambiguity aversion. *Journal of the European Economic Association* 7 (2-3), 649–658. DOI: 10.3386/w14713

Box, G.E.P., & Tidwell, P.W. (1962). Transformation of the independent variables. *Technometrics*, *4*, 531-550.

Buckert, M., Schwieren, C., Kudielka, B. M., & Fiebach, C. J. (2014). Acute stress affects risk-taking but not ambiguity aversion. *Frontiers in neuroscience*, *8*, 82.

Cameron, A.C., & Trivedi, P.K. (2005), *Microeconometrics: Methods and Applications*, Cambridge, Cambridge University Press.

Carver, C. S., & White, T. L. (1994). Behavioral inhibition, behavioral activation, and affective responses to impending reward and punishment: The BIS/BAS scales. *Journal of Personality and Social Psychology*, *67* (*2*), 319-333.

Ellison, P.T., Bribiescas, R.G., Bentley, G.R., Campbell, B.C., Lipson, S.F., Panter-Brick, C., & Hill, K. (2001). Population variation in age-related decline in male salivary testosterone. *Human Reproduction*, *17*, 3251–3253.

Elwood, C.E., Poythress, N.G., & Douglas, K.S. (2004). Evaluation of the Hare P-SCAN in a non-clinical population. *Personality and Individual Differences*, *36*(*4*), 833-843.

Ferrigan, M.M., Valentiner, D.P., & Berman, M.E. (2000). Psychopathy dimensions and awareness of negative and positive consequences of aggressive behavior in a nonforensic sample. *Personality & Individual Differences*, *28* (*3*), 527–538.

Gummelt, H.D., Anestis, J.C., & Carbonell, J.L. (2012). Examining the Levenson Self Report Psychopathy Scale using a graded response model. *Personality and Individual Differences*, 53 (*8*), 1002-1006.

Halevy, Y. (2007). Ellsberg Revisited: An Experimental Study. *Econometrica*, *75* (*2*), 503–536.

Harman, S.M., Miller, E.J., Tobin, J.D., Pearson, J., & Blackman, M.R. (2001) Longitudinal effects of aging on serum total and free testosterone levels in healthy men. Baltimore Longitudinal Study of Aging. *J. Clin. Endocrinol. Metab*., *86*, 724–31.

Levenson, M.; Kiehl, K.; Fitzpatrick, C. (1995). Assessing psychopathic attributes in a noninstitutionalized population. *Journal of Personality and Social Psychology*, *68* (*1*), 151-158.

Loomans, M.M., Tulen, J.H.M., de Rijke, Y.B., & van Merle, H.J.C. (2015). A hormonal approach to anti-social behavior. *Criminal Behavior and Mental Health*.

Marion, B.E., & Sellbom, M. (2011). An examination of gender-moderated test bias on the Levenson Self-Report Psychopathy Scale. *Journal of Personality Assessment*, *93* (*3*), 235–243.

McHoskey, J.W., Worzel, W., & Szyarto, C. (1998). Machiavellianism and psychopathy. *Journal of Personality & Social Psychopathy*, *74*, 192–210.

Pfattheicher, S. (2016). Testosterone, cortisol and the Dark Triad: Narcissism (but not Machiavellianism or psychopathy) is positively related to basal testosterone and cortisol. *Personality and Individual Differences*, *97*, 115-119.

Ross, S.R., Lutz, C.J., & Bailley, S.E. (2004). Psychopathy and the Five Factor Model in a Noninstitutionalized Sample: A Domain and Facet Level Analysis. *Journal of Psychopathology and Behavioral Assessment*, *26*(*4*), 213-223.

Rotter, J. B. (1966). Generalized Expectancies for Internal versus External Control of Reinforcement. *Psychological Monographs*, *80* (*1*), 1–28.

Smith, P.B., Trompenaars, F., & Dugan, S. (1995). The rotter locus of control scale in 43 countries: A test of cultural relativity. *International Journal of Psychology*, *30*(*3*), 377-400.

Stanton, S.J., Mullette-Gillman, O.A., McLaurin, R.E., Kuhn, C.M., LaBar, K.S., Platt, M.L., & Huettel, S.A. (2011b). Low- and high-testosterone individuals exhibit decreased aversion to economic risk. *Psychological Science*, *22* (*4*), 447–453.

Tackett, J.L., Herzhoff, K., Harden, K.P., Page-Gould, E., & Josephs, R.A. (2014). Personality × hormone interactions in adolescent externalizing psychopathology. *Personality Disorders*, *5*(*3*), 235–246.

Tversky, A., & Kahneman, D. (1991). Loss Aversion in Riskless Choice: A Reference Dependent Model. *Quarterly Journal of Economics*, *107* (*4*), 1039-1061.

Walters, G.D., Felix, C.M., & Reinoehl, R. (2009). Replicability and cross-gender invariance of a two-dimensional model of antisociality in male and female college students. *Personality and Individual Differences*, *46*(*7*), 704-708.

Yildirim, B.O., & Derksen, J.J.L. (2015). Clarifying the heterogeneity in psychopathic samples: Towards a new continuum of primary and secondary psychopathy. *Aggression and Violent Behavior*, *24*, 9-41.

Zaidi, I.H., & Mohsin, M.N. (2013). Locus of control in graduation students. *International Journal of Psychological Research*, *6*(*1*), 15-20.
